# Sensationalising the Female Pudenda: An Examination of Public Communication of Aesthetic Genital Surgery

**DOI:** 10.5539/gjhs.v5n2p153

**Published:** 2012-12-26

**Authors:** Ashong C. Ashong, Herbert E. Batta

**Affiliations:** 1 Department of Communication Arts, University of Uyo, Uyo, Nigeria

**Keywords:** decency, ethics, quality, honesty, balance, sensationalism, cost, language

## Abstract

We live in a society where beauty and sensations are important. Advances in medical technologies have brought on waves of new notions of beauty where commercial interests both in the media and the health industry spurred by fashion, advertising and celebrity promotion have tended to popularise body modifications and enhancements. In recent times, through offerings on cable television channels and glossy consumer magazines, medical procedures hitherto only in the precincts of medical schools, gyneacological clinics and medical journals have now pervaded the population. More seriously, on the Internet particularly, medical experts now offer services and graphic details of labiaplasty, clitoral hood reduction or enhancement, vaginal rejuvenation, etc. Here, we examine the public communication of the phenomenon of aesthetic genital surgery and interrogate thus; is it decent, honest, balanced and ethical? Relying on textual analysis, personal observation and literature review for data gathering, we observe that besides tending to commercialise and medicalise the female genitalia, a coalescence of medical, advertising and fashion interests as played out in the media sensationalises the benign science of plastic surgery and robs it of its truthfulness, genuineness, and purposefulness. The conclusion is that in Africa, where the effect of the development crises is telling, the hype surrounding cosmetic or aesthetic genital surgery is a damaging distraction particularly when the continent is waging a battle against female genital mutilation. The recommendations are that media and medical regulatory bodies should impress it upon media and medical industry operators that glaring commercial promotions of cosmetic genital surgery in the public media be checked, and that such communication should bear equal weight of facts related to risks, short comings, complications, and threats; in physical, social, and psychological terms.

## 1. Introduction

A natural take off point in a discourse such as this, is the definition of terms. Lets define the following terms:

*Sensationalism:* Generally speaking, this term is used to depict all aspects of media messages that are capable of attracting attention, exciting or inflamming emotions. It is related to terms such as commercialisation and tabloidisation (McQuail, 2005).

*Female Pudenda:* This term refers to the female genitalia. It covers parts such as the pubis, the vulva, the inner and outer labia, the clitoris and the vagina.

*Aesthetic Genital Surgery:* This refers to surgical (plastic) procedures performed on the female external genitalia for cosmetic reasons, i.e. to improve its appearance and function.

From the beginning of time, humans have always been concerned about their physical appearance. Batta (2011) writing on feminine aesthetics notes that women as well as men through the ages have modified, altered, adorned or enhanced their appearance by paying special attention to the hair, face, lips, ear/nose, eyes, neck, arms, abdomen, nails, skin, calf and more intimately; breasts, buttocks, and genitals. These were and are still done as seen in art, through the clarification, intensification, and interpretation – in one word – manipulation of the shape, texture, smell, and somatic speech or body language.

In recent years, the modification, adornment, alteration and enhancement of intimate body parts particularly the breasts, buttocks, and genitals through plastic surgery especially for cosmetic or aesthetic as opposed to medical reasons have been on the increase. The globalisation of mass communication, international trade, tourism, satellite broadcasting, mobile telephony not only continues to shrink the world but tends to create, foster, and spread a synchronous culture. To this effect, x-rated (pornographic) magazines depicting images of the female pudenda published in Euro-America become available to all parts of the world. Similarly, satellite broadcast programmes such as Dr. 90210 illustrating breast enhancement and labia reduction surgery using laser technology in sophisticated plastic surgery clinics in Beverly Hills, United States are viewed in homes even in remote parts of Africa. Also, thousands of websites and millions of web pages offered by the Internet and the World Wide Web containing graphic images of “before and after” photographs of cosmetic female genital surgeries, the surgeons who perform them, the cost of the surgeries, etc. are available for browsing by anyone in any corner of the world with access to a laptop, a personal computer, a cyber-café or a hand-held iPad, palmtop or mobile phone.

The import of this is that the ideas, facts, data, and pictures concerning aesthetic female genital surgery are in the public domain. Very significantly, the narratives, discourses, and controversies surrounding a related subject matter - female genital mutilation – a cultural and or religious practice common among Africans, Middle Eastern and some Asian countries are also in the public domain. Indeed, in scholarly writing, popular literature, and electronic broadcasting, the general theme has been that the practice represents violence to the female gender, damages the health of victims, reduces the depth of female sexuality, and is against human rights of women. In fact, recently, the British Broadcasting Corporation, World Service Radio as well as the Aljazeera satellite television have drawn attention to another related but dangerous practice among women in the Cameroons and the Congo – breast ironing. This involves massaging to flatten the breast with hot metal or wooden pestle in order to make the mammary organs less sensitive to touch and less attractive to men.

In this article, our preoccupation is to highlight the trend toward sensationalising or commercialising through medicine, the female genitalia. We examine the public communication of aesthetic female genital surgery particularly in the Internet and pose the following questions:

i) What is the nature and quality of aesthetic female genital surgery in the cyberspace?
ii) How honest or truthful is public communication of aesthetic female genital surgery?
iii) What is the motive of communicating aesthetic genital surgery in the cyberspace?
iv) How safe, fair, balanced and ethical is the communication of aesthetic genital surgery in the cyberspace?
v) What is the cost and implications of aesthetic genital surgery?
vi) How may suggestions or recommendations improve the communication of aesthetic genital surgery in the cyberspace?

## 2. Objectives

The objectives of this discourse are to:

a) Highlight the parallels between female genital mutilation and aesthetic genital surgery.
b) Explore the occurrence, nature, and quality of aesthetic female genital surgery in the cyberspace.
c) Examine the honesty or truthfulness of the communication of aesthetic genital surgery in the cyberspace.
d) Determine the motive for communicating aesthetic genital surgery in the cyberspace.
e) Find out how safe, fair, balanced and ethical the communication of aesthetic genital surgery is in the cyberspace.
f) Ascertain the cost and implications of aesthetic genital surgery.
g) Proffer suggestions that may improve the public communication of aesthetic genital surgery in the cyberspace.

## 3. Rationale

Several reasons account for the choice of the Internet as the focus of this discourse. As noted earlier, aesthetic genital surgery narratives occur in various media of communication – books, journals, magazines, newspapers, radio, television, cable and satellite broadcasting. We choose to preoccupy ourselves with its occurrence in the new medium – the Internet or the World Wide Web for the following reasons: The Internet according to McQuail (2005, p. 138) citing Poster (1999) integrates radio, film, and television and distributes them by means of “push” technology thus:

*It transgresses the limits of the print and broadcasting models by (1) enabling many-to-many conversations; (2) enabling the simultaneous reception, alteration, and redistribution of cultural objects; (3) dislocating communicative action from the posts of the nation, from the territorialised spatial relations of modernity; (4) inserting the modern/late modern subject into a machine apparatus that is networked*.

Other new and key medium attributes according to McQuail that make the Internet an attractive medium for study include: digitisation of all aspects, convergence of different media, Internet divergence from mass communication, adaptation of publication roles, greater “interiority” of the audience role, fragmentation and blurring of the “media institution” and reduced social control. Others are: interactivity, sociability, media richness, autonomy, playfulness, privacy, and personalisation.

## 4. Theoretical Framework

To the extent that the communication of aesthetic female genital surgery in the cyberspace bears marks of persuasion i.e. presenting information with a view to educating the audience on the facts and figures of cosmetic genital surgery and convincing people of its viability, such communication can be said to approximate advertising. After all, all the perquisites of advertising are present, namely: product, place, promotion, and price. Baran and Davis (1995) citing Shally (1987), and Hay (1989) view advertising as the ultimate cultural commodity. They add that,

*Advertising packages promotional messages so that they will be attended to and acted upon by people who often have little interest in and no real need for most of the advertised products or services. Consumption of specific products is routinely portrayed as the best way to construct a worthwhile identity… or solve problems (often ones you never knew you had (p.333)*.

Scholars have criticised the tendency of depicting a fixed (normal) image of female genital aesthetic and to drive the need to alter, modify, or enhance the female genitals through surgery to fit with this image.

Two theories can be said to be at work concerning the public communication of aesthetic female genital surgery: (a) value-expectancy theory and, (b) elaboration likelihood theory. Infante, Rancer, and Womack (1997) explain that value-expectancy theory as an approach to persuasion is based on the idea that the value we expect from something controls our attitude. What this means is that, if we value youthful looks, greater genital comfort, and increased sexual satisfaction as the consequences of aesthetic genital surgery, then we are likely to have a positive attitude to both the communicated message and the procedure. This is said to work at two levels: Affective – cognitive consistency operates thus: we have affect (attitude, feelings) and cognitions (beliefs, knowledge) regarding a topic and we try to make the two consistent. In other words, if we believe that good consequences will result from aesthetic genital surgery, we will favour (accept) it. The second level is learning theory where we learn to associate consequences with proposals. If a woman conditions herself to the knowledge that the vulva comes in various shapes, sizes, and colour and that every vulva is normal for the owner then she would not be persuaded to “improve” its appearance with cosmetic surgery. On the other hand, if a woman learns to associate pregnancy, childbirth, and old age with, “loose, flabby, uneven, unsightly, or wrinkled” vulva, she may likely find aesthetic genital surgery communication acceptable and aesthetic surgery a solution to the “problem.”

The elaboration likelihood theory first explained by social psychologists Petty and Cacioppo (1986) recognises that persuasion can result mainly from the attributes of the persuasion message or from the nature of the persuasion situation. Infante, Rancer, and Womack (1997, p. 171) not that, “when a persuasion message attempts to influence an attitude which the receiver realises is significant to his or her life, the likelihood increases that the receiver will cognitively elaborate on the content of the message”. What can be inferred from this is that, the more someone is personally involved in the subject of aesthetic female genital surgery, the more likely he or she will elaborate on the message on aesthetic genital surgery.

## 5. Method

This discourse is hinged on ideas, facts, and data gleaned from the textual examination of aesthetic genital surgery websites and a review of scholarly literature in books, academic journals, and Internet sources. The purpose of the analysis is to determine the intention, language, quality, honesty, and ethics of communicating aesthetic genital surgery information through the Internet. Printouts from the following top twenty websites obtained between the months of January and March 2012 formed the bases for the discourse:

(1) Manhattan Centre for Vaginal Surgery, New York, United States.
(2) Plastica.ca, Toronto, Canada.
(3) Millsmedical.com. United Kingdom.
(4) Urogyn.org at The Laguna Institute for Aesthetic Vaginal Surgery, Orlando, Florida, United States.
(5) Courthouse Clinics.com, London, United Kingdom.
(6) Labiaplasty Surgery.com, Virginia, United States.
(7) Clinic of Plastic Surgery, Brazil.
(8) Drmatlock.com at Laser Vaginal Rejuvenation Institute, Los Angeles, United States.
(9) Wellness Kliniek.com. Belgium.
(10) Cosmetic Surgery2.com, Florida, United States.
(11) Altermd.com Beverly Hills, California, United States.

## 6. Review of Literature

Writing on “Bodies of flesh, bodies of knowledge,” Maureen Whitcomb (2010) demonstrates that existing representations stress the differences and ignore the potential similarities that exist between female genital mutilation (FGM) and female genital cosmetic surgery (FGCS). Based on World Health Organisation (WHO) (2008) definition of female mutilation as, all the procedures involving partial or total removal of the external female genitalia, or other injury to the female genital organs for non-medical reasons, Whitcomb groups these procedures: reduction labiaplasty, vaginoplasty, liposuction to mons pubis, fat injections to labia majora or mons, clitoral hoodectomy, hymenorraphy, G-spot amplification and the use of surgical laser in vaginal rejuvenation as constituting FGCS. The prevalence of FGCS in the United States and the United Kingdom has risen to 30 percent, according to Whitcomb. She adds that for labiaplasty, the most popular procedure has an age range from adolescence, as young as ten-years old through to women in their 50s and 60s. Women in their 20s and 30s however are most predominant. Vaginoplasty, on the other hand is typically performed in older women who have given birth. She states further that there is insufficient documentation of both the safety and effectiveness of these procedures. Potential complications are said to include infection, altered sensation, dyspareunia, adhesions and tearing.

In attempting to trace the origins of female genital cutting, Whitcomb writes that the practice dates back to antiquity and was viewed as a sign of distinction, or a mark of enslavement or subjugation. It is also said to have resulted from the primitive man's desire to gain mastery over the mystery of female sexual function. Some believe that the practice has religious roots while others see it as playing a part in the patriarchal family system by ensuring and preserving male lineage. In framing female genital cutting as a functioning social and cultural convention, in some cultures, shame for the family may result from a woman's refusal of FGC. Also, where the female genitals are viewed as threat to men, FGC may be justified. In other societies, FGC becomes a means of connecting women with men through marriage as well as a means of protection from both aggressive men and from a woman's own sexuality. Besides, in some cultures FGC is seen as a beautification technique that enhances feminine genital aesthetics.

Looking at female genital cosmetic surgery as a functioning social convention, Whitcomb states that FGCS is performed for aesthetic, functional and psychological reasons. Female genital cosmetic surgeons tend to justify aesthetic genital surgery by describing the female genitalia as “irregular, misshapen, large, enlarged, fat, abnormal, problem, excess, deformed, asymmetrical, jagged, not smooth, flappy”. For these reasons, women's feelings towards their genitalia are said to cause, “embarrassment, loss of self-esteem, lack of confidence, and self-consciousness.” Before genital surgery, women's vulvas are more likely to be described as, “stretched, sagging, loose, aging, old, relaxed, gaping” whereas, after aesthetic genital surgery, the vulvas are described as “tightened, youthful, younger, renewed.”

Finally, Whitcomb believes that although female genital cutting and female genital cosmetic surgery both derive from social and cultural pressures to conform to ideals of beauty and femininity, there are major differences:

One major difference is the prevalence. Whereas the occurrence of FGCS is in the thousands … between 100 and 140 million women undergo FGC. Another major difference… is the idea of consent. In many cultures, FGC is performed on children from infancy to age 15…, whereas FGCS is often performed on consenting adults (p. 5)

The reasons for the recent mainstreaming of female genital cosmetic surgery include: marketing as seen in surgical reality shows, Internet pornography which offers a very narrow aesthetic of female genitalia, appearance of FGCS in public discourse through more clinical reports and media coverage in women's magazines, and increasing use of skimpy bikinis, thong underwear, Brazilian waxing, laser hair removal, oral sex, provocative fashion advertising and doctors who publicise techniques for beautification of the vulva and increasing sexual responsiveness.

These sorts of concerns according to Whitcomb prompted the New View Campaign to:

*Actively protest against FGCS because the practice is fairly unregulated and unmonitored … they exemplify the medicalisation of women's sexuality and the ways in which it creates new risks, negative norms, and insecurities. The campaign… emphasises the diversity of normal female genitalia and scrutinises FGCS for the pathologicalisation of female genitalia. This campaign, unlike what is presented by female genital cosmetic surgeons does not represent FGCS as an empowering, liberating practice that provides women with knowledge, choice, and alternatives. Instead, it depicts the practice as one that provides women with false information about the normality of diverse genitalia and their overall sexual health (p.13)*.

Writing on “Genitals and ethnicity: the politics of genital modifications,” Johnsdotter and Essén (2010) state that, “genital modifications take place in a sphere where biology, medicine and cultures are intertwined” (p.29). They add that, “female genital cutting/mutilation/circumcision has been discussed by Westerners for centuries, often as a bizarre and cruel practice far away in Africa,” but that, “there is a growing occurrence of procedures wherein Western women are having their genitals altered through surgery.”

The authors list these surgeries to include reduction of the inner labia, vaginal tightening, hymen “reconstruction”, clitoral “lifts,” and liposuction of the mons veneris but lament that little research has been carried out to map the prevalence and consequences of these procedures whereas there is a growing demand in the United States and British population. The rise of genital cosmetic surgery is said to show similarities to other trends where market forces and culturally based views of body and sexuality intertwine. In medical circles, it is observed that genital cosmetic surgery is often done without therapeutic necessity and that some of the practices are similar to those labeled female genital cutting.

Johnsdotter and Essén note that women who choose to modify, enhance, or alter their bodies in several ways may be seen as victims of patriarchy, beauty industry, the pressuring ideals of today or their inner insecurities. They further state that, “some may say that female genital cutting and genital cosmetic surgery are not comparable procedures … but if we disregard context and only focus on what in the anatomy is removed, the modifications are indeed comparable” (p. 32).

For the reason seen above, they suggest that, “scrutiny of the laws prohibiting female genital cutting in various Western countries makes it obvious that it is difficult to ban female genital cutting while condoning genital cosmetic surgery” (p.32). They conclude that, “if there can be serious consequences for sexual pleasure from cutting away the external female genitals, then surgical excision practices among European women also need to be challenged on those grounds” (Johnsdotter & Essén, 2010, p. 34).

Delving into, “Self-regulated cosmetic surgery in the United Kingdom: a poor prognosis for autonomy,” Latham (2010) observes that cosmetic surgery is increasingly popular in the UK – moving up from 10,738 surgeries in 2003 to 34, 453 in 2007. These operations are done in private clinics in response to patients’ request for aesthetic enhancement of their bodies and not for medical reasons. Latham notes that, “female patients still outnumber male patients by a ratio of 9 to 1 and that women are, “oppressed by the growing culture of cosmetic surgery than men” and that because these procedures involve invasive surgery, “patients run the risks of blood loss, bruising, infection, deep vein thrombosis, wound healing problems, scarring, even death” (p.48).

Private sector advertising and marketing, Latham (2010) writes, may also increase false expectations amongst patients. Also, patients are unlikely to have been referred by their general practitioners, self referred people may not disclose important contraindications, are not on waiting lists, and this leave no time to reflect on the treatment or even reconsider it. These may lead to disfigurement and injury. It is important that patients seek adequate information on cosmetic surgery because there is a risk that the patient might fail to achieve her physical aspirations. She may also have psychological needs that are not investigated by the surgeons and thus place her mental health at risk if she becomes dissatisfied or requires more corrective treatment. For these reasons, it is necessary for patients electing to go for genital cosmetic surgery to note that their care could be less than adequate, there could be lack of written guidance on clinic procedure, misleading advertisements about the potential success of treatment, lack of information about practitioners’ qualification and inadequate medical record and clinical governance (Latham, 2010).

Concerning activism on the medicalisation of sex and female genital cosmetic surgery, Tiefer (2010) x-rays the activities of the New View Campaign in the United States. The New View Campaign (NVC) is a grassroots initiative which challenges the over-medicalisation of sex and the construction of female sexual dysfunction and offers alternative clinical formulations to deal with women's sexual problems. According to Tiefer (2010, p. 57), the NVC has equally looked into how contemporary sexuality is a special market. To Tiefer, “sex enhancement obviously offers great financial opportunities, but poor sex education and the moral contests involved in public discourse about sexual pleasure have created an ignorant and vulnerable public”.

Reporting further on the activities of NVC, Tiefer states that one such activity targeted surgeons who performed female genital cosmetic surgery, who advertised online and through the media, claiming sexual benefits from FGCS, and posted before-and-after genital photographs on their website. The Campaign bore messages to the effect that, more research and less marketing are needed regarding female sexuality: Other messages were:

-Vulvas are beautiful, variegated and no two are alike;
-Female genital surgeries have consequences;
-The Food and Trade Commission should ban ads promoting cosmetic genital surgery;
-Long labia are normal and desirable;
-Women should keep their vulvas away from the surgeon's knife;
-Women should oppose the marketing of designer vaginas;
-Genital cosmetic surgery guarantees no satisfaction;
-There is need for more scientific research and none for advertisements promoting violence against female genitals (Tiefer, 2010, p. 59).

Writing on “Norms and bodies; findings from field research on cosmetic surgery in Rio de Janeiro, Brazil, Dorneles de Andrade (2010) observes that cosmetic surgery among others are forms of body modification that have parallels with the discourse on female genital mutilation and should be considered to have an analogous function in the reproduction of social and gendered objects. This author comments that, “while some authors see FGM as a manifestation of the abuse of women in a male-dominated system of ideals and norms of beauty, they criticise the rejection of FGM by those who at the same time accept cosmetic surgery in Western countries. Others do not disagree that FGM can have negative consequences for women's health but argue that social customs like FGM should not be treated as pathologies because the people whose customs are under attack will not share such an analysis” (p.75). She sees the decision of the individual to use cosmetic surgery as an expression of agency or autonomy and self-determination regarding a person's own body.

Dorneles de Andrade reports that Brazil has the highest number of cases of cosmetic surgery per capita in the world, 63 percent of Brazilian women were reported to have said they would like to have cosmetic surgery (the largest of any national group), that the profile of the “natural” vs., the “perfect” female body represents the role-model and comes from pornographic depictions of the female body and that the construction of “normality” vs, “deviance” or “normal”, vs. “abnormal” regarding female genitals and their surgical alteration is culturally shaped in the discourses of aesthetic medicine and the media. Dorneles de Andrade states that three different types of female genital cosmetic surgery were being conducted namely: the reduction of the labia minora, the filling or replenishing of the labia majora, and the narrowing/tightening of the vagina. On the reason for this practice, de Andrade (2010, p.78) states:

*Psychological problems such as an “inferiority complex” gave cosmetic surgery a therapeutic rationale. The idea of striving for happiness by recreating oneself, which emerged in 19th century philosophy of enlightenment (still) represents the justification for cosmetic surgery*.

She lamented her inability to find published scientific research conducted in Brazil on female genital surgery but found a large number of advertisements in magazines and newspapers claiming the advantages of “intimate” surgery and comments thus:

*The cult of body has become a mass phenomenon and taken on an important social dimension in an information society where norms and images are broadcast worldwide by the media. Self-realisation of the individual through body-modification… proceeds, and for a growing number of people, the body is becoming the locus of self expression and self-determination (p. 81)*.

The author notes that there is a great need for further studies of the consequences of cosmetic surgery for physical and mental health and that the belief that both female genital mutilation and female genital cosmetic surgery are harmful practices for women's psychological and physical health is a step towards a process of rethinking and reconceptualising individual, societal, and cultural ideals and norms.

Echoing the view of cosmetic surgery as deeply reductive as it speaks directly to the fantasy of reinventing the self, Andrades is saddened by what is happening to medical ethics in relation to cosmetic surgery because with the growth in a consumer culture, ethics in medicine is becoming more bendable and subject to the law of the market. This view tallies with that Plowman (2010, p. 113) who reechoes the argument that, “much of the success of female genital cosmetic surgery is because its promoters created the problem – that the privacy of this part of the body can foster anxiety, playing on the long standing cultural taboo surrounding female sexuality – which was then sold to women alongside the solution of surgery”. It also means that the creation of a market for female genital cosmetic surgery, Plowman agrees, is for providers’ financial gain rather than a solution to a pre-existing unresolved problem. This is very suggestive of why Berer (2010) calls for the regulation of cosmetic surgery:

*Cosmetic surgery is not well regulated and much has been left to self-regulation. Cosmetic surgery requires… oversight and regulation. Thorough investigation is needed of many aspects of labia reduction and other types of genital modification surgery; including who is carrying it out and where, the quality of care in these facilities and level of fees charged, how and where the practitioners are being educated and trained, what if any benefits the procedures have for physical or mental health and for quality of life and relationships and whether the procedures are being promoted in an unethical manner by playing on women's poor self-image and lack of confidence about their looks, and fears of not being able to find a partner or enjoy sex because of the shape of their labia. The views of (young) men and the role they play in their partners seeking cosmetic genital surgery, as well as the role of the media, are equally important to investigate. On the basis of this evidence, policy and regulation should be developed*.

## 7. Findings

### 7.1 The Cyberspace and the Characteristics of Aesthetic Female Genital Surgery Communication

The Internet contains a plethora of information on cosmetic genital surgery. For example, google search for “top cosmetic genital surgery sites” we conducted on the 4th of October, 2011 yielded 8,870,000 results. However, for the purpose of this study, only eleven (11) such sites were purposively selected. We included sites owned by aesthetic female genital surgery clinics in the United States, Europe, Canada, and Brasil.

### 7.2 Manhattan Centre for Vaginal Surgery (http://www.Centreforvaginalsurvery.com). New York, United States

The rationale behind aesthetic female genital surgery to this organisation is, “society's increasing acceptance of woman's expectations of sexual satisfaction and happiness…” It continues, “many women are embarrassed… about vaginal looseness, enlarged or irregular labia or loss of sexual satisfaction.” It performs labiaplasty often for, “cosmetic enhancement or medical reasons. Vaginoplasty is done to “increase friction during intercourse that often enhances sexual satisfaction” while vaginal rejuvenation, combines labiaplasty and vaginoplasty to, “yield cosmetically enhanced labia and, tightened vagina during one surgical visit.” The centre equally performs hymenoplasty to “repair torn hymen for religious or cultural reasons.” The site encourages visitors to, “see actual results” of “before and after surgery photo gallery of actual patients…”

### 7.3 Clinic of Plastic Surgery, Brazil (http://www.clinic-brazil)

In this website, “intimate plastic surgery, corrects dysfunctions and improves the woman's hidden aesthetics”. It divides cosmetic vaginal surgery into three groups: “Vaginoplasty (mainly vaginal tightening and muscle re-building, Labiaplasty (reducing and/or reshaping the external structures, and Hymenoplasty (re-construction of the hymen, to a “near-virginity state.”

The website adds that, “women with large labia can experience pain during intercourse, or feel discomfort during everyday activities or when wearing tight fitting clothing. Others may feel unattractive, or wish to enhance their sexual experiences by removing some of the skin that covers the clitoris. Furthermore, the website states, “many women dislike the large protuberant appearance of their labia minora. This may cause severe embarrassment with a sexual partner.” The only word of caution is that, “after surgery, women may experience some mild discomfort and swelling,” but then; “this disappears completely after 1-2 weeks” and that labial incisions usually heal and are rarely noticeable. To cap it, the site also advertises the cost of intimate surgery it performs. Vaginoplasty costs 850 euro, labia minora reduction goes for 650 euro; labia majora reduction is done for 650 euro while hymenoplasty costs 350 euro. These exclude the cost of anaethesia and hospital stay.

### 7.4 Wellness Kliniek (http://www.wellnessklinick.com)

Based in Belgium, this clinic performs various forms of cosmetic surgery including labia reduction, labia argumentation, hymen repair, vaginal rejuvenation, etc. The website regrets that, “for ethical reasons we can’t show you before and after photographs on our website. We do however want to inform you about the costs”. The site states that labia reduction costs 1960 euro, labia argumentation costs 1475 euro, while vaginal rejuvenation goes for as high as 6950 euro.

### 7.5 Female Genital Surgery, Toronto Clinic (http://www.plastica.ca)

This website promotes, “Dr. Martin Jugenburg's years of experience performing vaginal rejuvenation, labia reduction and hymen reconstruction procedures ensure you will receive the most advanced treatment available.” The site also states that labia reduction surgery trims protruding labia minora which, “causes discomfort during sexual activity…” or becomes unsightly.” It warns that labiaplasty carries potential risks to include: bleeding, infection… wounds, and asymmetry between the right and left labia.

### 7.6 Labiaplasty Surgeon.com (http://labiaplastysurgeon.com)

This centre, based in Virginia, United States features Dr. Bernard Stern as, “a leading expert in cosmetic surgery with over 25 years of experience,” encourages Internet surfers to read Dr. Stern's vaginoplasty patient case study, view his patients’ photo gallery and read Dr. Sterns’ patient testimonial. The site states: “many women dislike the large protuberance of their labia minora. This may cause severe embarrassment with a sexual partner or loss of self esteem and in some cases pain and discomfort.” It continues: “Not long ago, labiaplasty was usually only performed within a select group of entertainers and performers – women such as swimsuit models and centrefold models. But today, with the advent of more sexually permissive magazine/videos, apparel and behaviour, the importance of genitalia are much more prevailed. Most often, labiaplasty is being done for two reasons… medical… and aesthetic… aesthetic reasons (beautification) are being driven by societal evolution, regarding sexual habits, wants and expectation. This is the primary reason driving the market for genital surgery.” The cost of aesthetic female genital surgery is said to vary depending on complexity of repair but typically ranges from $1850 to $10,500.

### 7.7 Courthouse Clinics (www.courthouse clinics.com)

This UK-based female genital surgery clinic states that, “the most common reason that women generally request this procedure is that the labia are perceived to be too large and aesthetically unpleasing.” The surgery is said to take one hour and it makes them, “feel more confident in intimate situation and happier with the way they look”. The prices of these surgeries are said to start from £2950.

### 7.8 Mills and Mills Medical Group (http://www.millsmedical.com)

According to this group, female genital surgical procedures “are becoming increasingly common in the UK and worldwide.” It adds that, “you may have read about or seen TV programmes on the subject in the media” and that, in the media, it is sometimes referred to in short-hand as, “designer vaginas.” This site offers free consultation and advice and bears description of the procedures as well as pre-and post-operative requirements for labia reduction, vaginal tightening and reshaping, and hymenoplasty. The site also includes before and after photos of the female genitals.

### 7.9 Cosmetic Surgery, P. A. (http://www.cosmetic surgery2.com)

This centre is based in Palm Beach, Florida, United States. It states that “how a woman “perceives” how she looks in the vaginal region can have devastating effects on her life. It can threaten her self-esteem, reduce her sexual excitement, ruin her love life or cause vaginal discomfort.” This website touts Dr. Bernard Stern, its surgeon as having, “experience, knowledge and sensitivity that make him the perfect person to perform… vaginal cosmetic surgery.” On the website, visitors can immediately see graphic pictures of the female vulva demonstrating the benefits of vaginal cosmetic surgery in their anatomical detail but warns visitors to proceed, “only if the material will not offend” them. The site urges visitors to call their office to book for a free consultation or complete an online contact form. The rationale for this surgery, the site maintains, is to “not only recreate more youthful and aesthetically pleasing external genital structures, but to restore self-image and self esteem.”

### 7.10 The Languna Institute for Aesthetic Vaginal Surgery (http://urogyn.org)

This institute is based in Florida, United States. It states in its website that it specialises in restoring and enhancing the appearance of the vaginal area because, “the loose and unsatisfying feeling that women feel can also be felt by their male partner during intercourse.” It further states that, “labial enlargement, unevenness… makes it look unappealing.” This site contains surgery photographs, and promotes Dr. Alinsod, its vaginal aesthetician, as having, “the distinct advantage and experience as a reconstructive pelvic surgeon.” Moreover, since Dr. Alinsod is not like a plastic surgeon who lacks training in this sort of surgery, or the gynecologist who has little training in vaginal aesthetics, he possesses aesthetic, gynecologic, and urologic qualifications that make him, “the surgeon of choice.”

### 7.11 Gary J. Alter (MD) (http://www.utermd.com)

Alter is based in Beverly Hills, New York. According to his website, “physicians have neglected aesthetic surgery of the female external genitalia. However, awareness of female genital aesthetics has increased owing to increased media attention, from both magazine and video publications. Women may feel self-conscious about the appearance of their labia majora, or more commonly, labia minora. The aging female may dislike the descent of her public hair and labia. A large pubic fat may be unsightly.” To “correct” these problems, Gary Alter offers operation in labia reduction, clitoral hood reduction, pubic liposuction and lift, vaginal rejuvenation, etc. on the website, complete with before and after photos.

Additionally, several aesthetic female genital surgery websites bear testimonials from satisfied clients. Hardly do we find testimonies of unsatisfied ones. Here are some examples:

(a) (a)“One patient, a 22-year-old college student from Toronto, said she had never had intercourse until after her labiaplasty because she felt “insecure and ugly” about excess labia tissue” (http://www.labiaplastysurgeon.com).
(b) “Dr. Matlock is a genius who is precise and a perfectionist at what he does. This is cosmetic art as I perceive it and requires extreme talent and intelligence. I recommend this surgery to women who feel different in their insecurity…” (http://drmatlock.com/testimonials.html).

## 8. Discussion

### 8.1 Quality, Honesty and Beauty in Aesthetic Female Genital Surgery

In summary then, from the examination of the communication of aesthetic female genital surgery on the Internet as illustrated above, the following can be said to characterise the public communication:

a) With regard to *decency*, it can be said that the public communication of aesthetic genital surgery in the Internet is characterised by graphic details of the female genitalia to the extent that a few websites warn visitors that the images could offend their sensibilities. Most websites offer no such caution. This sort of display may border on prurience.
b) In terms of ethics, most of the websites bear characteristics that tend toward unethical practices. There is evidence of soliciting for clients, offering of loans and freebies, giving incomplete information, fueling insecurities about vulva aesthetics, and advertising of physician credentials, skills, and services. Moreover, the service is largely unregulated or at best self-regulated.
c) Concerning the *quality* of information, accuracy is sacrificed. The impression the websites give is that aesthetic genital surgery is desirable even if there are no medical indications. Again, one set the impression from the websites, that there is an analogous relationship between the much despised female genital mutilation common in third world countries and female genital cosmetic surgery common in the developed world.
d) A focus on *honesty* indicates that it may not be the welfare of the women folk that drives the female aesthetic genital surgery industry but financial fortunes for plastic surgeons, the fashion and modeling industry, the pornographic magazine and film sectors as well as the new age view of the pseudo feminine aesthetic.
e) In examining *balance*, it is apparent that there is a lot of lopsidedness in the communication of aesthetic female genital surgery. The testimonials are more often than not positive – praising the virtues and benefits of genital surgery to the woman, applauding the skills and services of genital surgery experts and down playing on the risks, complications, and potential harm that aesthetic genital surgery may cause.
f) Furthermore, the public communication of aesthetic female genital surgery is characterised by *sensationalism*. A lot of hype surrounds the issue not only in the Internet but in the press, the broadcast media, cable and satellite systems as well as in public conversation. The media tend to offer cosmetic female genital surgery as entertainment. The more scientific and correct view is that the female genitals come in various lengths, widths, colours and shapes and if they are not damaged by trauma such as occur in vesico-vaginal fistula, difficult and multiple births or old age; there is absolutely no need for surgery or a “designer vagina.”
g) In relation to *cost*, the communication of aesthetic genital surgery in the Internet loudly indicates that though the procedures are touted as simple, requiring local anaesthesia, and an outpatient treatment, the cost seems too high, prohibitive and tends toward financial exploitation. The websites show that aesthetic female genital surgery ranges from 350 to 6950 euro or between 1850 and 10,500 US dollars. That might be seen as a waste on a needless surgery.
h) On the positive side, considering *language*, it could be said that the communication of aesthetic female genital surgery is characterised by simple-to-understand diction though some difficult terms e.g. “protuberant,” etc. are used. Generally speaking, a person of average education can read and understand the language.
i) Similarly, in terms of website/webpage aesthetic, it can be said that the postings use good aesthetic. The colours are inviting, the photographs (apart from those that show graphic anatomical details of the female genitalia) are alluring – beautiful, smooth female faces, well nourished bodies, etc. (though this is make-believe) and the typography appropriate. However, some of the websites carry too much information as to make the pages cluttered.

The observations that we have made above following the textual examination of the communication of aesthetic female genital surgery have been corroborated by several other scholars. Arroba (2003) sees it as fallout of the patriarchal system which is based on the control and appropriation of women's bodies – movement, attitudes, sexualities, fertility, life cycles, pregnancies, and births, menopause and aging and overall health by “experts.” Writing on the medicalisation of women's bodies in the era of globalisation, Arroba states that, “one third of women's lives are marked by aging, one-third of our bodies are fatty tissues, and both… have been transformed into surgically – correctable problems.” She concludes that, “behind it all is profit – it's all about money in the end. The cosmetic surgery industry is a billion dollar industry. In order to guarantee their income, plastic surgeons distort women's self-perception and magnify their self-hatred and rejection. We see it every day in the media, the magazines, medical brochures, television, the movie industry, the newspaper, and advertising…” (p. 2).

In the same vein, Blanchard (2010) reviewing Marge Berer's article notes how the female genitalia are described as, “too big, too small, too narrow, too wide, too high, too low, too flabby, and too wrinkled. These permutations are endless. What a great way of making money” (p. 2). She further notes that, surgery has penetrated the beauty industry and has become a high earner with a powerful and growing influence. She laments that whereas there are laws in Europe and Africa against female genital mutilation surgery, none exist against female cosmetic surgery. She adds her voice to the fact that few women really understand the risks, complications and outcomes of these surgeries and that cosmetic surgery deserves a close look, better regulation and that the whole notion is profit driven and manufactured.

In line with our observation on ethics, Lee (2011) gives statistics on how big the designer vagina business is: in 2009 female consumers spent an estimated $ 6.8 million on the procedures. It is equally popular in the UK. In 2008, there were 1,118 labiaplasty operations, 70% more than the previous year as well as over 5000 inquires about cosmetic gynaecology in 2010 and 65% about labial reduction and the rest for tightening and reshaping the vagina. Lee states that, “in the US, cosmetic gynaecology may have the official sanction of reality TV … but the same cannot be said of the peer organisations. The American Congress of Obstetricians and Gynaecologists, (ACOG) deems such procedures medically unnecessary, possibly unsafe and is, “concerned with the ethical issues while the accrediting body, the American Board of Obstetrics and Gynaecology (ABOG), refuses to recognise cosmetic-gyn as a legitimate sub-specialty,” (p.2).

Still on ethics, Bates (2010, p.1) states that cosmetic labial surgery has been at the centre of escalating controversy pitting critics against proponents in a battle of words over ethics, evidence-based medicine, and philosophical questions of free choice and societal pressure and concludes that, “doing such procedures and advertising them with photographs of purportedly “attractive” versus “unattractive” genitalia constitute a violation of the ethical relationship gynaecologists have with their patients… To Bates, women should be educated about the wide range of normal vulva anatomy and it is not right for gynaecologists to exploit patients’ psychological vulnerabilities merely by offering aesthetic procedures since the decision implies an endorsement of aesthetic deficiencies among normal women.

## 9. Conclusion and recommendations

There is no better way to end this discourse than doing so in the words of Goodman (2009, p. 154):

*Genital plastic surgery for women has come under scrutiny and been the topic of discussion in the news media, online, and in medical editorials. In the absence of measurable standards of care, lack of evidence-based outcome norms and little standardisation either in nomenclature or training requirements, concern has been raised in both ethicists and specialty organisations. Some women request alteration of their vulvas and vaginas for reasons of cosmesis, increasing self-esteem, and improving sexual function. Patients must be assured their surgeon is properly trained and should understand that few validated long term safety or outcome data are presently available in this relatively new field, women also should be made aware that, although they may wish to cosmetically or physically alter their external genitalia, this does not mean that they are developmentally or structurally “abnormal”. It is important that training guidelines for practitioners be established and that long term outcome, psychosexual and safety data be published. The genital plastic surgeon must have sufficient training in sexual medicine to withhold these procedures from women with sexual dysfunction, mental impairment, or body dysmorphic disorder*.

In addition, the code of medical ethics by the American Medical Association issued June 2003 provides a befitting guide or recommendation with information that is helpful to mass communicators, online website and page hosts, genital aestheticians, and the consuming public. For, in the words of Goodman (2009, p.159) patients and others need to know and take note of the ethical principles of autonomy (acting completely autonomously, free from outside influence, and devoid of mental impairment, depression, anxiety…), non maleficence (any procedure that has a greater chance of causing harm than good is unethical), beneficence (ethical obligation of the physician to promote the health and welfare of the patient), justice (the resources of society are used to the greater good of society), and veracity (truth telling, is important in surgical counseling and decision making).

Therefore, the AMA Code relating to the Use of Health-Related Online sites is as follows:

i) Physicians who establish or and involved in health-related online sites must minimise conflicts of interest and commercial biases. This can be achieved through safeguards for disclosure and honesty in funding and advertising. It also requires that physicians not place commercial interests ahead of patient health; therefore physicians must not use health-related online sites to promote unnecessary services, refer patients to entities in which they have ownership interests, or sell products outside of established ethical guidelines.
ii) Physicians who establish or are involved in health-related online sites that use patient specific information must provide high level security protections, as well as privacy and confidentiality safeguards.

In the light of the above and in view of the online sites examined in this article it is clear that there is glaring non compliance. While it is true that the practice of aesthetic female genital surgery is not rampant in the developing world, it is true that people there do take medical tours abroad for cosmetic genital surgery, and have online agencies that scout patients for Western-based aesthetic genital surgeons. It is also true that there is a long standing practice of using herb-based products to purportedly enlarge the breast, penis, vulva and buttocks as well as those that tighten the vagina. Whether they are efficacious or not is open to study. It is therefore important that public communicators in print, broadcast, film, and online media have adequate knowledge of these issues so that the public can be better informed and educated.

To this end, we recommend that media regulators take a more than cursory look at the communication of aesthetic female genital surgery online to ensure that they conform to journalistic standards of honesty, truth, accuracy, fairness, balance, quality and decency. Similarly, medical regulatory authorities should closely monitor the practice and communication of aesthetic female genital surgery to encourage it to operate in accordance with medical principles and ethics of autonomy, non-maleficence, beneficence, justice, and veracity.

In doing these, the quality, honesty, and beauty in aesthetic female genital surgery communication would become the ideal that we all desire. Most importantly, it is desirable that the practice and communication of aesthetic female genital surgery by studied, investigated, monitored and regulated in order not to send to places where female genital mutilation is practiced, the signal that female genital mutilation is desirable, harmless, and beneficial.
